# Supplementary material for: The Yeast Ribosomal Protein Rpl1b Is Not Required for Respiration
Source: Int J Mol Sci. 2024 Oct 28;25(21):11553. doi: 10.3390/ijms252111553 (PMC11545921; doi:10.3390/ijms252111553)
Supplement: Supplementary file 1 [file ijms-25-11553-s001.zip › ijms-3174541-supplementary.pdf]

Supplementary Figures 1, 2, and 3

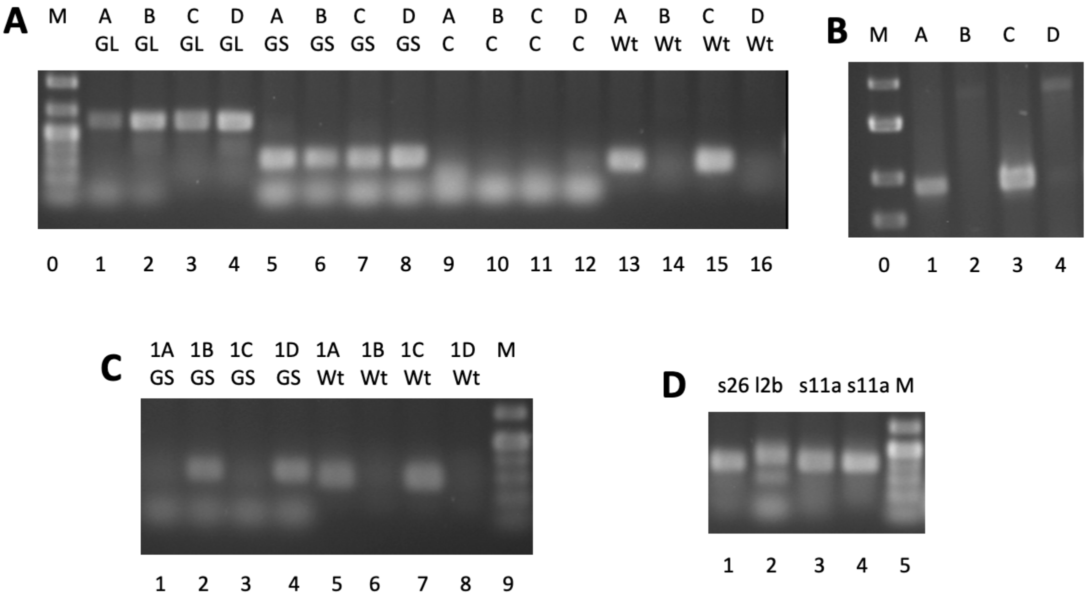

Fig. S1. PCR Characterization of Strains.

A. Segregation of the *KANR::rpl1b* knockout in BF534 tetrad 12.

The four spore clones (A, B, C, and D) from tetrad 12 were analyzed by PCR for the *KANR::rpl1b* knockout using two different primer pairs (lanes 1 to 8) and for the wild-type *RPL1B* gene (lanes 13 to 16), and with a negative control (lanes 9-12). Each lane is labelled with the name of the spore clone (A, B, C, or D) and the primer pair used for PCR amplification (see Materials and Methods for primer sequences). The “GL” pair is “G418 Long”, and consists of a forward primer from the 5’ UTR of RPL1B, and a reverse primer inside the G418 ORF, generating a PCR product specific for the deletion. The “GS” pair is “G418 Short”, and consists of the same forward primer from the 5’ UTR of *RPL1B* as above, paired with a reverse primer from the G418 cassette closer to the beginning of the cassette, generating a shorter deletion-specific fragment. The “C” pair is a “Control” pair, and consists of primers with a Tm too low to function under the conditions of this PCR. The “Wt” pair is “Wild-type”, and consists of the same forward primer from the 5’ UTR of RPL1b as above, paired with a reverse primer from just inside the *RPL1B* ORF. See Supplementary Fig. 2 for a map of the locations of primers in genes of interest. “M” is a marker from New England Biolabs.

B. Segregation of the wild-type *RPL1B* gene in BF534-12.

The same PCR reactions as in lanes 13-16 of panel A, but re-run on a higher percentage agarose gel for improved resolution.

C. Segregation of the mutant and wild-type *RPL1B* genes in BF535 tetrad 1.

Like panel A, but assaying the strains BF535-1A, 1B, 1C, and 1D. 1B and 1D score as rpl1b::G418 by PCR, and are also the two G418-resistant spore clones. The fastest-migrating band in lanes 1-4 is primer or primer-dimer.

D. PCR analysis of the *rps26* ("s26"), *rpl2b* ("l2b"), *rps11a* (MATa) ("s11a"), and *rps11a* (MATalpha) ("s11a") mutants from the deletion collection. The primers used are analgous to those used for *KANR::rpl1b* in Fig. 1. For each of the three genes, the forward primer comes from the 5' UTR of the gene, and the reverse primer is G418 Short. The PCR products seen are specific to the deletion alleles.

Figure S2: Tetrad Segregation for a Disome

Cross: Chr. VII *RPL1B* x Disomic Chr. VII *rpl1b::G418*
rpl1b::G418

Parental Ditype Tetrad (1 in 6)	Non-Parental Ditype Tetrad (1 in 6)	Tetratype Tetrad (4 in 6)
RPL1B	*RPL1B, rpl1b*	*RPL1B*
rpl1b, rpl1b	*rpl1b*	*rpl1b*
RPL1B	*RPL1b, rpl1b*	*RPL1B, rpl1b*
rpl1b, rpl1b	*rpl1b*	*rpl1b, rpl1b*

Tetrad 534-12D is a non-parental ditype with two *rpl1b::G418* spores, B and D. All four spores are resistant to G418, and all four contain *rpl1b::G418*. But spores A and C also contain the wild-type *RPL1B*.

Fig. S3: Map of PCR Primer Locations

5’ region of *RPL1B*

GCACTTCTTGA**GCATTACGTTATTTGGTAACCTC**TTGAAGTTTCAGGAAAGACTCCATAG
AACTAGTCGCAAGCCTCACGGACCACCAAATACTTTGGAAGACTAATTACATATCATAAA
ATGTCTAAGATCACCTCTTCTCAAGTCAGAGAACACGTCAAGGAATTGTTGAAGTATTCC
AACGAAACCAAGAAGAGAAACTTCTTGGAAACCGTCGAACTACAAGTCGGTTTGAAGAAC
TATGACCCTCAAAGAGACAAGCGTTTCTCT**GGTTCTTTGAAGTTGCCAAAC**TGTCCAAGA
CCAAACATGTCCATCTGTATCTTCGGTGATGCTTTCGATGTTGACAGAGCTAAGTCTTGC
GGTGTTGACGCTATGTCCGTCGATGACTTGAAGAAGTTGAACAAGAACAAGAAGTTAATC

280 bp PCR fragment, “Wt”, primer pair “Wt”, RPL1b upUTR F and RPL1b ORF R,
GCATTACGTTATTTGGTAACCTC and GTTTGGCAACTTCAAAGAACC.

The 5’ region of *RPL1B*, including part of the 5’ UTR, and part of the open reading frame. The initiator ATG is shown in bold. The sites of PCR primers are shown in bold.

5’ region of *rpl1B::G418*

GCACTTCTTGA**GCATTACGTTATTTGGTAACCTC**TTGAAGTTTCAGGAAAGACTCCATAG
AACTAGTCGCAAGCCTCACGGACCACCAAATACTTTGGAAGACTAATTACATATCATAAA
ATGcgtacgctgcaggtcgacggatccccgggttaattaaggcgcgccagatctgtttag
cttgcctcgtccccgccgggtcacccggccagcgacatggaggcccagaataccctcctt
gacagtcttgacgtgcgcagctcag**gggcatgatgtgactgtcgc**ccgtacatttagccc
atacatccccatgtataatcatttgcatccatacattttgatggccgcacggcgcgaagc
aaaaattacggctcctcgctgcagacctgcgagcagggaaacgctcccctcacagacgcg
ttgaattgtccccacgccgcgcccctgtagagaaatataaaaggttaggatttgccactg
aggttcttctttcatatacttccttttaaaatcttgctaggatacagttctcacatcaca
tccgaacataaacaaccat**gggtaaggaaaagactcacgttt**cgaggccgcgattaaatt
ccaacatggatgctgatttatatgggtataaatgggctcgcgataatgtcgggcaatcag

274 bp PCR fragment, “G418 Short”, primers RPL1b upUTR F and KanR R short,
GCATTACGTTATTTGGTAACCTC and GCGACAGTCACATCATGCCC.

571 bp PCR fragment, “G418 Long”, primers RPL1b upUTR F and KanR R long,
GCATTACGTTATTTGGTAACCTC and AAACGTGAGTCTTTTCCTTACCC

The 5’ region of the *rpl1B::G418* deletion construct from the yeast deletion set. Sequences originating from *RPL1B* are in upper case; sequences from the KanMX4 module are in lower case. The initiator ATG of *RPL1B* is in bold. The sites of PCR primers are shown in bold.
